# Extensive Laceration of the Liver and Diaphgram, from the Kick of a Horse

**Published:** 1848-09

**Authors:** E. C. Banks

**Affiliations:** Lawrenceville, Illinois


*Extensive laceration of the Liver and Diaphragm, from the kick of a horse.* By Dr. E. C. Banks, of Lawrenceville, Illinois.

Some time in the spring of 1844, I was called, in haste, to see a boy aged seven years and some months, son of a blacksmith, of this place. He had been kicked by a horse, and immediately became so bad, that his parents, who were summoned by the lady at whose house he was, thought it advisable to let him remain for a while, and have a physician immediately to see him, which they did. When the boy came to the house of the lady, he asked her for a bed, and said a horse had kicked him in the side, and placing his hand at the same time over the liver, said it hurt him very much, and that he wished his parents who lived in an adjacent block, to be sent for, which was immediately done. They came, and called in a young physician who then resided in the place, who applied stimulants externally and internally. I found the patient's pulse scarcely perceptible at the wrist, and very quick; extremities cold, cheeks red, and he was very restless, casting himself about from side to side, and swearing the bitterest oaths—language which he was not accustomed to employ in health. He manifested symptoms of pain, but could not give any information to us how and when the accident had happened, or where his distress was seated. Stimulants were applied,—warm brandy toddy internally, and warm bricks to his feet and hands, and also fomentations of hot water and brandy and Cayenne pepper applied by frictions to the extremities. Toddy given at intervals, so as to allow the same to be checked whenever the slightest appearance of reaction was perceived. The parents had me called shortly after the accident, thinking I would bleed him, as the other physician had refused to do so, and very properly. Finding, in spite of all our exertions by powerful frictions and warm applications externally, and toddy internally, that he was sinking, we gave him a warm bath, and kept him in it about twenty minutes. Took him out and wrapped him in warm blankets, continuing the toddy. Finding all means fail, and suspecting internal hemorrhage and a fatal termination, the family were informed of our apprehensions, and accordingly they had him placed on a bed and carefully carried down to their own house, only fifty yards distant. Just as they were going into the house he expired.

On a post mortem next day, (this being about sundown) it was discovered that the liver was torn half across, just about the middle of it. His abdomen was literally filled with blood, as likewise his chest. Yet there was no mark, either internal or external, on the parietes of the abdomen, of any injury. Turning up the right lobe of the liver, we found the diaphragm ruptured to a wonderful extent, making an opening that you could put your double fists through. The blood had passed from the abdomen into the chest. The rent in the liver was so large that one could bury their two fists in it. It is proper to mention that the horse from which the kick was received, was without shoes. It seems extraordinary that the kick of a horse, without making any external mark, should tear the liver half in twain, and at the same time make such a rent in the diaphragm.

*December 25th*, 1847.

